# 

**Published:** 1879-07-20

**Authors:** 


### *EDITORIAL NOTICES.*

☞ *WANTED.*—All the money that is due us on subscriptions. Friend, your good intention does not enable us to pay the printer. He requires the cash. Please remit at once.

*Decennial Pharmacopeal Convention.*—The Sixth Decennial Pharmacopeal Convention is appointed to meet in Washington, D. C., on the first Wednesday in May, 1880.

*Wm. R. Warner & Co.'s Quinine Pills.*—We have been furnished with a beautiful sample of sugar-coated quinine pills from the house of Wm. R. Warner & Co. They are put up in superb style, and are pure and excellent. These sugar-coated pills we can recommend as pure, soluble and reliable, as we have tested them in practice, and know whereof we speak. See their new advertisement in this number.

*Colden's Liebig's Extract of Beef*, the advertisement of which may be seen in our journal, is doubtless an excellent preparation. The need of safe and invigorating dietetic preparations in low conditions of the system is almost daily felt by the practitioner. The above preparation is certainly worthy of trial, its value having been tested and sustained by eminent practitioners.

*Southern Medical College Announcement.*—The great interest which seems to be felt by the profession in this new Institution, and the numerous inquiries made in regard to it, has induced us to insert the College Announcement in our Miscellaneous Department. The corner-stone of the building was laid by the Masons on the 8th instant, with appropriate and inspiring ceremonies, with music, and in the presence of a large concourse of citizens. The address of Dr. T. S. POWELL, President of the Board of Trustees, and that also of Rev. D. E. BUTLER, Grand Master and conductor of ceremonies, were both highly commended as master-pieces of eloquence. Numerous articles were deposited in the corner-stone by different individuals, the list of which we regret we have not space for at present. It will be noticed that the Chairs of *Surgery* and *Chemistry* are not yet supplied. We are assured the Board will see that they are properly and ably filled, and will soon announce their names. We trust that this new and important Southern enterprise will secure the approval and influence of every member of the profession. W.

RECEIPTED.—1878: H. J. Anderson; J. W. Benton. 1878 and 1879: M. W. Eason; J. F. Earnest. 1879: W. Carpenter; C. M. Green; P. E. Thomas; J. B. Ingraham; S. W. Eaton; A. R. Oglesby; A. V. Ponder, 6 months; A. Atkinson, 6 months; W. H. Gautier; H. M. Lawson; S. H. Anderson; W. H. Wilson; Thos. J. Hendley. 1878: W. M. Guice.

